# Clinical Evaluation of Patients with Vestibular Dysfunction

**DOI:** 10.1155/2019/3931548

**Published:** 2019-02-03

**Authors:** Vijay Renga

**Affiliations:** Dartmouth Hitchcock Medical Center, Geisel School of Medicine at Dartmouth Lebanon NH, 03766, USA

## Abstract

Dizziness is a common reason for outpatient neurology consultation. Oftentimes, a complete workup by general practitioner, including MRI brain fails to reveal a cause. Some patients would have also undergone an ENT evaluation before approaching neurology for an answer. Such scenarios provide a challenge as well as opportunity for the neurologist to exercise their knowledge and clinical skills in arriving at a diagnosis. Conditions like ‘Unspecified Vestibular Dysfunction' and ‘Presbyvertigo' are often the underlying causes, which are either not recognized or misdiagnosed as BPPV, psychogenic or perceptive dizziness. This article's goal is to help understand vestibular system and diagnose vestibular dysfunction in clinical practice.

## 1. Introduction

Clinical neurology has seen profound changes during the past couple of decades with the advent of MRI. However, some conditions that will not show up on an MRI or a myriad of testing can still be diagnosed by the knowledge and skills of a neurologist. Dizziness is the classical one.

### 1.1. Clinical Scenario

A 78-year-old male with history of atrial fibrillation, hypertension, diabetes, and coronary artery disease is referred for evaluation of dizziness. The patient had recurrent episodes of dizziness and falls. Most of those falls happened when he was bending over, turning around, or climbing stairs. During ED visits and follow-ups with the primary care physician, there was concern of TIA. An MRI of brain was obtained which showed nonspecific white matter lesions. Echocardiogram showed left ventricular hypertrophy and right atrial dilatation. EKG revealed atrial fibrillation. A carotid ultrasound showed bilateral 50-59% narrowing. He was referred to vascular surgery with a concern of TIA. Vascular surgery did not think that his dizziness was due to the carotid disease and referred him to neurology for further evaluation.

Which of the following is the most likely cause of his dizziness?

- Carotid disease
- Vertebral artery disease
- Cardiac arrhythmia
- Orthostatic hypotension
- Vestibular dysfunction

Given the title of this article, most readers would have guessed the right answer. Rest of this article explains why it is so.

## 2. The Balance System

Our balance system is activated whenever there is a concern for movement. It is active not only when someone is moving but while maintaining a balanced posture, for example, as in standing on a straight line. Once that person lays on a bed, closes their eyes, and tries to avoid any movement, the system becomes dormant again.

Oftentimes, when you ask a patient with dizziness if they have ever had symptoms while lying still in a bed, they would say, “no, not until I twist or turn or try to get up”. The diagnosis could then be narrowed down to a balance system dysfunction.

**Figure 1:**
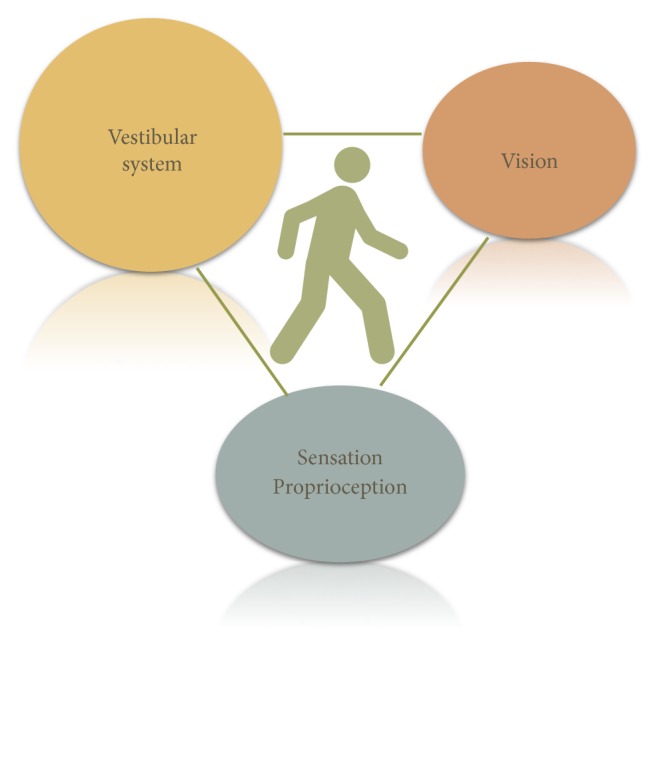
**The Balance System.** Balance as a function of vestibular system with visual and sensory inputs.

So, what is the balance system? The balance system that we are talking about is the vestibular system with inputs from visual and sensory pathways, Figure 1.

## 3. Vestibular System

When used in common parlance, the vestibular system refers to the inner ear and its connections. But, that only refers to the peripheral vestibulocochlear apparatus which is the main receptor of the vestibular system and not the vestibular system per se. At the same time, the majority of pathologies within the vestibular system lie within the vestibule. Therefore, it is important to further understand this receptor.

## 4. The Peripheral Vestibular Apparatus

The vestibulocochlear apparatus has two parts, the vestibule and the cochlea. The vestibule is involved with balance and cochlea with hearing. Let us ignore the cochlea in this discussion, while acknowledging the fact that the biggest clue to a peripheral vestibular dysfunction is ‘hearing loss', since they are two halves of the same organ.

The vestibular apparatus has three bony labyrinths within which lies the membranous labyrinth. The bony canals contain perilymph which communicates with CSF though the cochlear aqueduct and has a composition similar to CSF with high sodium and low potassium. CSF pressure can get transmitted to perilymph and patients can experience dizziness with CSF pressure fluctuations.

Suspended within the bony labyrinth and floating in the perilymph is the membranous labyrinth. This delicate structure follows the three-dimensional contours of the bony labyrinth. The membranous labyrinths end in a dilated ampulla and communicate with the utricle and saccule which are known as the otolith organs. The membranous labyrinth is filled with endolymph which unlike the perilymph has low sodium and high potassium. It does not communicate with the CSF and is a closed compartment, but is susceptible to changes in external milieu. Endolymph pressure changes in the system are delicately maintained. Medications like diuretics and agents like alcohol can cause changes in the composition of perilymph and endolymph causing labyrinthine dysfunction and imbalance.

The membranous labyrinths dilate into ampullas within which are located highly specialized ‘hair cells'. The hair cells convert a wave impulse into a neural impulse. These hair cells are probably the most critical and most fragile part of the vestibular system and can be affected by age, infection, medication, and metabolic processes. By the age of 80, around 40 percent or less of hair cells remain, unless they have already been damaged by infections or other causes [1, 2]. Hair cells in the ampullae are embedded in a tuft of crista ampullaris and detect fluid movements. They are also present in the otolith organs in a different arrangement to capture effects of gravity and linear motion. Hair cells are innervated by the distal branch of bipolar cells in the Scarpa's ganglion, the proximal part of which forms the vestibular nerve. When hair cells are mechanically bent, impulses travel to the Scarpa's ganglion and then to the vestibular nerve and vestibular nuclear complex in the brainstem. Signals may be excitatory or inhibitory based on the direction of bending of hair cells [3]. The continuous discharge from the hair cells maintains a vestibular tone [4].

## 5. The REAL Vestibular System

**Figure 2:**
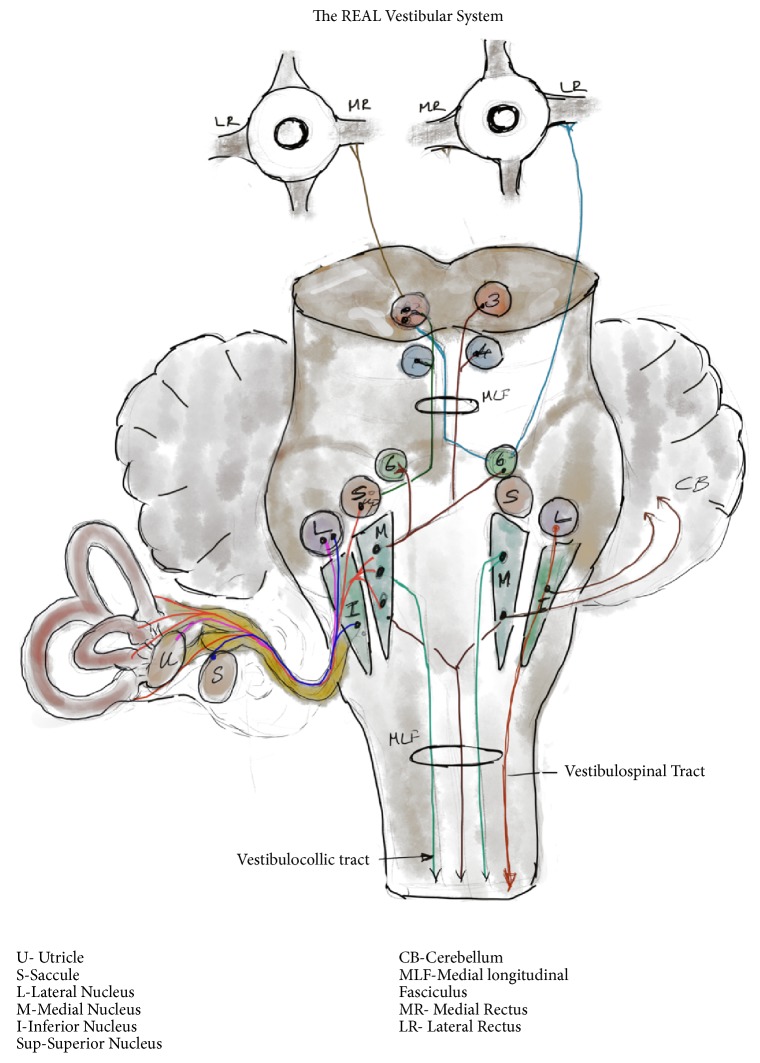
**The real vestibular system.** Impulses are carried from the horizontal semicircular canals and the otolith organs to the vestibular nuclei. Ascending and descending pathways that travel to various cranial nerve nuclei III, IV,VI are depicted here. Few fibers go directly to the cerebellum without synapsing in the vestibular nuclei. Ascending fibers travel through MLF to control ipsilateral and contralateral eye movements. Descending pathways travel to the neck and trunk through the vestibulocollic and vestibulospinal pathways to control neck and body positions to maintain balance.

As stated earlier, the vestibulocochlear apparatus is not the vestibular system. It is only a receptor, but the most important one for the vestibular system. The vestibular system is a very complex network intricately linked to most cortical and subcortical structures. A large proportion of neurological problems can be explained if there is an understanding of the vestibular system. So, let us take a look at the its components, Figure 2.

### 5.1. Afferents

The inner ears or the vestibular apparatus forms the major input. Proprioception, visual signals, and muscle proprioceptors provide additional input. Neck muscles give significant input, which is underrecognized.

### 5.2. Center

The inputs primarily reach the vestibular nuclear complex in the pontomedullary junction and the cerebellum. There are 4 major vestibular nuclei: superior, lateral, medial, and descending, and some minor nuclei [5]. The superior and medial nucleus are relays for vestibuloocular reflex. Medial nucleus also provides inputs to the vestibulospinal and vestibulocollic pathways. Lateral nucleus is primarily connected to the vestibulospinal tracts. The nuclei and pathways are interconnected ipsilaterally and contralaterally which helps in coordination and synchronization of movements. They are also connected to the cerebellum which allows for coordination and fine-tuning. Some relays from the vestibule also go directly to the cerebellar nuclei bypassing the vestibular nuclei. Relays to and from cortex and subcortical structures modulate voluntary and involuntary components of balance system. Most reflexes mediated by vestibular system are involuntary and much faster than voluntary control.

### 5.3. Efferents

There are 3 main efferents for this system:

*Vestibuloocular pathways* serve the vestibuloocular reflex, which controls the eye movements in response to head movements.

*Vestibulocollic pathways* serve the vestibulocollic reflex, which acts on the neck muscles to make neck adjustments in relation to head positions.

*Vestibulospinal pathways* serve the vestibulospinal reflex, which controls the truncal posture in response to head movements

In addition, the* vestibulo-autonomic pathways* are connected to the autonomic nervous system [6].

## 6. The Role of Vestibular System

The main function of vestibular system is to

- adjust head, neck, and trunk position necessary to maintain balance;
- maintain images at the fovea with head and neck movements.

## 7. Mechanism of the Vestibular System

The vestibular system's role is achieved through three reflex mechanisms [7].

### 7.1. Vestibuloocular Reflexes (VOR)

VOR helps maintains images on to fovea. As the head turns to follow an object, the eyes have to move proportionally in the opposite direction to maintain the target at the fovea. The horizontal canals respond to angular motion. The saccule and utricle respond to linear acceleration and gravity: the saccule senses acceleration in the sagittal plane and the utricle senses acceleration in the horizontal plane. Head rotation along a plane causes variable fluid shifts in the ipsilateral and contralateral semicircular canals along that plane. The right and left horizontal canal, the right posterior and left superior canal, and the right superior and left posterior canal form coplanar pairs. Along with the otolith organs, they sense motion in almost any plane.

The superior vestibular nerve carries impulses from the utricle to the vestibular nucleus which travels through the medial longitudinal fasciculus to the oculomotor nucleus. It stimulates the oculomotor nucleus and the medial rectus and inferior oblique. An electrode placed below the eye could capture this potential as a phasic response called ocular Vestibular Evoked Myogenic Potential (oVEMP). The inferior vestibular nerve carries impulse from the saccule to the medial vestibular nucleus. The vestibulospinal tract carries these impulses to the spinal accessory nucleus which innervates the sternocleidomastoid. A potential called cervical Vestibular Evoked Myogenic Potential (cVEMP) can be obtained by recording the SCM muscle [8].

The cerebellum is involved in the central modulation and regulation of vestibular function. Flocculus helps in VOR adaptations. Cerebellar modulus adjusts the duration of VOR responses by processing the otolith inputs and avoids under- or overshoot. Anterior superior vermis of cerebellum controls the VSR and gait and truncal stability [9].

To understand this better let us follow what happens during a head turn to the right side with eyes closed. The two horizontal canals act as the major coplanar pairs with variable input based on the tilt from the other canals. As the head turns to the right side there is shift of endolymph to the right on ipsilateral side and less so on the contralateral side. The right side would have a greater stimulation which is proportional to the velocity of the head turn whereas there is reduced discharge from hair cells on the left side. These impulses are transmitted through the superior vestibular nerve to the medial and superior vestibular nuclei and cerebellum. The signal ascends up through the ipsilateral and contralateral pathways through the MLF to the ipsilateral medial rectus and the contralateral lateral recuts causing the eyes to move to the left side. Therefore, the eyes move for the proportion, degree, and speed to the left side similar to a doll's eye. The cerebellum fine- tunes the process.

### 7.2. Vestibulospinal Reflex

VSR maintains body position in relation to head movements. Three pathways are involved in this function:

#### 7.2.1. Lateral Vestibulospinal Tract

It originates from the lateral vestibular nucleus and gets inputs from otoliths and cerebellum. It controls the antigravity muscles which maintain the extensor tone of the lower extremity muscles.

#### 7.2.2. Medial Vestibulospinal Tract

It originates from medial, superior, and descending vestibular nuclei and mediates postural and head righting responses. It controls the neck musculature.

#### 7.2.3. Reticulospinal Tract

It receives input from all vestibular nuclei and other sensory and motor systems which coordinates balance. It is involved in auditory, visual, and tactile balance.

Let us have another example, this time when head is tilted to the right side by someone walking a tightrope. Both horizontal canals and the otoliths are stimulated. Impulses are carried to the vestibular nucleus as described earlier. From the centers, the signals travel down through the medial and lateral vestibulospinal tracts to the spinal cord. Extensor activity is produced on the side to which head is tilted and flexor activity is induced on the opposite side.

### 7.3. Vestibulocollic Reflex

With the above description we have also covered the vestibulocollic reflex. This reflex maintains neck musculature in relation to head position. When the head is tilted to the right side as described above, the right SCM has to relax and the opposite side contracts.

## 8. Accessory Mechanisms

In addition to the major vestibular efferent reflexes there are accessory mechanisms that provides feedback and feedforward mechanism to maintain balance. These are the cervical reflexes, oculovestibular reflexes, somatosensory reflexes, and higher cortical and subcortical integration.

### 8.1. Cervical Reflexes

One of the most important and mostly underrecognized part of the vestibular system is the role of the neck. Extensive afferent and efferent pathways going to and from the neck serve the balance system [10]. Neck muscle dysfunction and tightening constitute a part and parcel of vestibular dysfunction. They could stem from an involuntary tightening of neck as a reflex mechanism by the vestibular system which also controls the bodily tone. The mechanism may be helpful for adaptation in the short term by reducing fluid shifts inside the vestibule, but ends up being counterproductive in the long term as it impairs most cervical reflexes needed to maintain balance.

*Cervico-ocular reflex* is a feedback type reflex controlling eye movements modulated by neck proprioceptors, which can supplement the VOR.

*Cervicospinal reflex* refers to the changes in limb position driven by neck afferent activity. The reticulospinal system plays a role along with the vestibulospinal system in maintaining this [7]. This causes extension of limb on the side ipsilateral to chin turn and flexion contralaterally.

*Cervicocollic reflex* is an intrinsic mechanism that mediates a neck reflex intrinsically from neck movements.

These reflexes could fail in patients with tight neck from arthritis or other causes and lead to imbalance.

### 8.2. Oculovestibular Reflexes (OVR)

OVR are the reflex mechanism occurring in response to eye movements or visual inputs from external environment. Visual inputs are the second major afferents for balance system. They have a longer latency than the vestibular reflexes but it becomes the major compensation for balance compromised by vestibular dysfunction. Vestibular imbalance worsens when patients are in the dark or when they close their eyes during washing their face or in a shower. Elderly patients falling in their bathrooms are often misattributed to slipping when in fact it may be due vestibular pathology.

### 8.3. Somatosensory Reflexes

Tendon receptors, sensation from feet, and motor impulses all contribute to the coordination from sensory inputs. In neuropathy, these inputs are diminished causing imbalance. Patients usually end up with wide ataxic gait for compensation. When there is a severe neuropathy of the peripheral nervous system, often there is involvement of the hair cells and vestibulocochlear nerve which results in hearing loss and balance problems.

### 8.4. Higher Integration, Cortical and Subcortical Control System

Thalamic relays to higher centers control vestibular functions. Higher processing is a complicated subject and not well defined. Various mathematical models try to explain it, but that is beyond the scope of this article [11].

There is high intrinsic variability and sensitivity of vestibular system in the general population. Some people are able to enjoy video games, virtual reality games, riding a bus, or being on a boat while others are throwing up.

## 9. Disorders of the Vestibular system

This article's purpose is just to touch upon the common conditions affecting the vestibular system. Vestibular dysfunction can be peripheral or central in origin. See Table 1.

Vestibular dysfunction is an umbrella terminology for a variety of conditions impairing the inner ear or its higher connections. Due to lack of imaging or electrodiagnostic tests to clearly identify and localize the specific process, many patients end up being diagnosed as psychogenic dizziness or perceptual dizziness, when in fact they have a mild nonspecific vestibular dysfunction that can be identified clinically. The article's main purpose is to highlight three underrecognized causes of imbalance.

### 9.1. Unspecified Peripheral Vestibular Dysfunction

 In author's experience, a unilateral or bilateral** Unspecified Peripheral Vestibular Dysfunction** is the commonest cause of dizziness seen in the outpatient settings. The peripheral vestibular dysfunction could be from a prior infection, trauma, medications, or a variety of other causes. Often it is mistaken with BPPV. In contrast to BPPV, there is no stone or bone problem. Nystagmus cannot be elicited and the Epley maneuver will not fix it. There are no imaging or electrical tests to detect it, but there are ways to diagnose it clinically.

### 9.2. Presbyvertigo

Like any other organ, vestibular system is susceptible to aging. As people grow older, their hearing diminishes, which is termed presbyacusis. The same process affecting the vestibular system is called “presbyvertigo”. This is probably the most common cause of vertigo in the elderly. There is limited literature on this condition.

### 9.3. Cervicogenic Dizziness

Another condition that remains largely unrecognized but debated is cervicogenic dizziness [12]. A pliable neck is a key factor for the cervical reflexes to work. Neck movement limited by arthritis, trauma, or neck spasm is a likely cause of dizziness [13].

Once we have acknowledged the above three conditions, we shall move on to the commonly recognized pathologies.

**BPPV** or Benign Paroxysmal Positional Vertigo is due to broken otoconia (stone) from the utricle dislodged into the endolymph, which interferes with the normal endolymph dynamics. Usually, this stone is lodged in the dependent posterior canal and leads to dizzy spells related to position which starts abruptly and subsides quickly. If dizziness is associated with a change in head position, this can be tested positive with nystagmus on a Dix Hallpike maneuver and responds well to Epley therapy.

**Meniere's disease** is where there is increased endolymphatic fluid in the system. The high pressure in the system causes dysfunction in hearing and balance.

In** superior canal dehiscence** there is erosion of the superior bony canal causing erratic pressure system in the canals and therefore dizziness.** Perilymphatic fistula** is a similar condition where there is abnormal communication between the inner and middle ears

**Vestibular migraine** often is a diagnosis when no other cause is found. However, the diagnosis should be made with caution as it has specific criteria described in ICHD-III [14, 15]. Migraineurs have a heightened sensitivity of vestibular system which could explain some of the sensitivity during testing, but a lack of association with migraine headaches and unilateral findings can often help exclude vestibular migraine as a cause.

Vascular causes usually lead to sudden severe dizziness which gets better over 24-48 hours. Almost always, dizziness is accompanied by dysarthria, ataxia, weakness, and sensory symptoms with involvement of extra-vestibular system and long tract signs.

Dizziness from presyncopal and hypotensive events could be from a reduced perfusion of the brainstem and vestibular structures causing failure of vestibular function.

## 10. Symptoms of Vestibular Dysfunction

Unilateral dysfunction typically causes vertigo. Bilateral dysfunction is perceived as lightheadedness or imbalance. Oscillopsia is due to bilateral dysfunction. Generally, in peripheral lesions vertigo predominates. Double vision, vertical nystagmus, or limb ataxia suggest a more proximal lesion at the level of brainstem or cerebellum

Hearing loss, tinnitus, and ear fullness often occur on the side of the peripheral lesion. Vision problems are common in vestibular dysfunction. There can be fluctuation in visual acuity and oscillopsia. These problems would have often been evaluated by ophthalmologists with no abnormalities detected.

Most patients with vestibular dysfunction end up with a tight neck. This is probably due to maladaptive mechanism as described earlier. It can lead to chronic headaches.

Chest tightness, sweating, palpitation, and other cardiovascular symptoms occur from involvement of vestibulo-autonomic pathways, which sends the evaluating physician on the wrong trail.

## 11. Clinical Evaluation of Vestibular Dysfunction

Numerous tests have been described for evaluating the vestibular system [16]. This article will highlight the most useful ones in clinic settings.

A vestibular system examination begins with a hearing test. A finger rub testing is good enough. Ears have to be tested individually as well as simultaneously. Oftentimes, this is when patients realize that they have a reduced hearing on one side.

### 11.1. Eye Movements

It important to look for nystagmus as it is a tell-tale sign. End gaze nystagmus is a normal phenomenon. Unfortunately, it looks like the nystagmus of a peripheral vestibular dysfunction except that the corrective phase is faster towards the normal side in vestibular dysfunction. Using a phone camera would be useful to review under slow motion. People with vestibular dysfunction generally get some dizzy symptoms when following a target to the affected side.

Spontaneous nystagmus occurs because of loss of tonic input from affected labyrinth. Peripheral nystagmus may have a torsional component which can make it look slightly vertical. Nystagmus from a peripheral lesion can decrease or stop if patients are asked to fixate their eyes on a target. Vertical nystagmus is always due to a central cause since those signals, compared to peripheral, run through a different tract to third nerve nucleus [6].

Head thrust test is a useful test. The patient is asked to fixate their eyes on a target, usually the examiner's nose. Examiner does a quick head turn of around 20 degrees to throw the eyes out of the fixated target, which would be countered by a normal vestibuloocular reflex. A displacement off target to the affected side with a catch-up saccade is an indicator of vestibular dysfunction [17].

Ocular tilt reflex tests the integrity of utriculoocular reflex but is difficult to elicit clinically.

Dix Hallpike maneuver is the classic maneuver described to detect a posterior canal BPPV. It causes nystagmus with the head hanging down or when switching to upright position from lying down. This test is specific for posterior canal BPPV which precipitates the nystagmus and dizziness. A more useful approach which I suggest and the readers can verify is a “vestibular shake-up using Dix Hallpike type maneuver” with a rapid, forceful elevation aimed at shaking up the whole vestibular system. The rapid forceful movement of the neck precipitates the typical dizziness in most patients, even if there is no nystagmus. The caveat is that it would precipitate dizziness in case of peripheral vestibular dysfunction or cervicogenic dizziness. Differentiating one from the other would be the next step.

### 11.2. Dynamic Visual Acuity

It can be done for both vestibular and cervicogenic processes. The idea is to detect changes in visual acuity after a vestibular shake-up or a cervical twist. Patients are asked to read from a Snellen's chart to check their best baseline visual acuity. After a head shaking test that visual acuity is checked again. If there is new impairment or dizziness, it is likely to be due vestibular dysfunction [18]. On the other hand, if the visual acuity changes or patient gets dizzy while the vestibular system is kept steady by supporting the patient's head and then having the body around the head for a neck torsion test, it is likely to be cervicogenic [19].

Sensory examination is important to detect neuropathy. The most useful test for vestibular dysfunction perhaps is a Sharpened Romberg test or a Tandem Stance test with the patient standing toe to heel, with arms crossed and eyes closed. The balance system is dependent on our visual and sensorimotor inputs to the vestibular system. In this test, we take out the components of vision and proprioception with closed eyes and narrow base with crossed arms when balance entirely depends on the vestibular system. There is a consistent sway or fall on to the affected side in unilateral peripheral vestibular dysfunction. With bilateral dysfunction there is imbalance and shaking with a tendency to fall to either side. Sway occurs due to the loss of ‘vestibular tone' on the affected side which is a hair cell function.

### 11.3. Other Tests

Modifications of the above testing can be done by using a foam pad under the feet to impair joint and proprioception receptors or to do a “Stepping test of Fukuda” [20]. In a stepping test, patient is asked to march with their eyes closed and arms straightforward. In unilateral dysfunction there is a tendency to turn to the side of vestibular dysfunction.

## 12. Laboratory Testing of Vestibular Function

Laboratory and electrodiagnostic tests for vestibular function are useful, but often limited as they are negative in about half of the cases of peripheral vestibular dysfunction [21]. Tests may be negative despite patients becoming symptomatic during the test or having clinical signs on examination.

Electronystagmography is the most readily available and commonly used test. Eyes act as a dipole with retina being negatively charged and the cornea being positive. When the eye ball moves towards the electrode, it detects a positive shift and negative when away. Eye movements are captured using an ENG machine. Nystagmus, its velocity, frequency, amplitude, and latency can be measured, even if eyes are closed. A video goggle added on can also detect and record minute eye movements making it Videonystagmography (VNG), which is more useful.

Bithermal caloric test is often used to test the horizontal canal function. Temperature fluctuations can induce endolymph shifts causing hair cell activation. This is usually done using warmed or cooled air towards the end of VNG testing.

Traditional VNG testing only evaluates superior branch of the vestibular nerve and angular vestibuloocular reflex pathway. It does not test the functions of the utricle and saccule which are done by specific tests for otolith function [8].

## 13. Tests of Otolith Function

### 13.1. Otolith Reflex Function Tests

These are myogenic potentials recorded as part of vestibular reflexes [8, 22, 23].

### 13.2. VEMP

Combining VEMP testing with VNG testing gives information about both the superior and inferior division of vestibular nerve. The otolith organs receive a low frequency stimulus causing fluid shift in the vestibule endolymph. This is a different frequency than hearing and works even in hearing impaired, given that the transmission of the sound waves to inner ear is not impaired by middle ear effusions, otosclerosis, etc. If the air conduction is difficult, bone conduction can be used.

### 13.3. cVEMP

Cervical VEMP responses are obtained from ipsilateral SCM muscle and measure sacculocollic reflex. Comparing the contralateral side is a good measure of intraindividual variability [8].

### 13.4. oVEMP

Ocular VEMPs are obtained from contralateral inferior oblique muscle after stimulation of the ipsilateral utricle [8, 22].

Superior semicircular canal dehiscence gives a high amplitude VEMP response of around ten times that of normal due to the reduced resistance to stimulation provided. Meniere's disease typically shows reduced cVEMP and oVEMP.

However, these tests are not well validated and the reliability goes down in elderly, the population in which vestibular dysfunction predominates.

## 14. Management

When peripheral system is dysfunctional, a vestibular suppressant medication like an anticholinergic, an antihistamine, or a benzodiazepine can help to reduce the symptoms [24]. Side effect is sedation, since the drugs cannot specifically target the vestibular system. It is better to avoid medications unless patient is having a bad spell. Even then, it is best limited to 3 to 4 days by which time symptoms resolve. Meclizine has limited efficacy, but benzodiazepines are effective for most patients.

Even though the vestibular system can get deranged easily, it has robust neuroplasticity [25, 26]. The networks can be trained back to a good level of functionality by Vestibular Rehabilitation Therapy [27]. Even with unilateral vestibulectomy, the body can adapt well as long as visual and proprioceptive pathways are intact. The adaptability is so good that often it is difficult to elicit vestibular dysfunction in a well-compensated patient.

The patient in the case scenario improved after VRT.

## Figures and Tables

**(a):** 

**Peripheral causes**

(i) Unspecified, including presbyvertigo
(ii) Cervicogenic dizziness
(iii) BPPV
(iv) Meniere's disease
(v) Vestibular Neuronitis/Labyrinthitis
(vi) Superior Canal Dehiscence
(vii) Vestibular migraine
(viii) Concussion
(ix) Acoustic neuroma

**(b):** 

**Central causes**

(i) Multiple sclerosis
(ii) Atypical Parkinson (PSP/MSA)
(iii) Stroke
(iv) Migraine
(v) Cerebellar disorders
(vi) Concussion
(vii) Medications
(viii) Psychogenic
(ix) Cardiovascular/Autonomic

PSP: progressive supranuclear palsy; MSA: multiple system atrophy.
